# Assessing Road to Mental Readiness (R2MR) training among correctional workers in Canada

**DOI:** 10.1186/s40352-023-00206-z

**Published:** 2023-01-23

**Authors:** Matthew S. Johnston, Rosemary Ricciardelli, Maryam Ghodrati, Stephen Czarnuch

**Affiliations:** 1 Fisheries and Marine Institute, Memorial University of Newfoundland, 155 Ridge Road, St. John’s, NL A1C 5R3 Canada; 2 Fisheries and Marine Institute, Memorial University of Newfoundland, W3023, 155 Ridge Road, St John’s, NL A1C 5R3 Canada

**Keywords:** Road to Mental Readiness (R2MR), Stigma, Mental Health, Correctional Workers, Public Safety Personnel

## Abstract

**Background:**

Mental health frameworks, best practices, and the well-being of public safety personnel in Canada are topics of increasing interest to both researchers and organizations. To protect and improve worker mental health, different training programs have been implemented to serve this population. The Road to Mental Readiness (R2MR) training regimen is one such program specialized to build cultural awareness of mental health, reduce stigma, and mitigate the cumulative impacts of exposures to potentially psychologically traumatic events among public safety personnel. However, limited research has been conducted to evaluate the effectiveness of R2MR, especially among correctional workers.

**Methods:**

The current study analyzed 307 open-ended survey responses to four (4) questions about R2MR garnered from 124 Canadian provincial and territorial correctional workers between 2018–2020 to reveal their understandings and perceptions of R2MR training, and to identify what learned skills they found challenging or easy to implement.

**Results:**

The results suggest that R2MR training plays a significant role in decreasing stigma and increasing mental health awareness. Across jurisdictions, R2MR creates a supportive space for open dialogue around mental health meant to shift cultural and individual barriers that often hinder treatment-seeking. Some respondents also indicated that R2MR was a starting point for intervention.

**Conclusions:**

Further research is necessary to understand how R2MR and other programs could support the mental health and well-being of correctional workers.

## Introduction

The Road to Mental Readiness (R2MR) mental health training and education program was initially developed by Canada’s Department of National Defence at the request of the Chief of Military Personnel and Canadian Armed Forces Surgeon General. The objectives of R2MR remain to help armed forces members achieve better mental health outcomes throughout their careers as well as improve their mental health awareness (Blackler et al., 2018). Key components of the R2MR training regimen include increasing mental health literacy, teaching stress management skills, and changing negative attitudes towards mental health service use, with the goal of lowering psychological distress among participants, increasing psychological resilience, and improving treatment-seeking when mental health challenges arise (Blackler et al., 2018). More specifically, the R2MR program “provides evidence-based psychoeducation on mental health stress…as well as providing a series of evidence-based cognitive behavioural therapy style skills designed to help participants to manage stress; for example, goal setting, mental rehearsal/visualization, adapted cognitive monitoring (i.e., awareness of self-talk), and arousal management through adapted breathing (i.e., tactical breathing)” (Carleton et al., 2018a, 2018b, p. 510). R2MR training involves diverse modules, but tends to be offered in one day or three day formats and often in person (at least prior to the COVID-19 pandemic and resultant public health measures). The program is delivered by trained public safety personnel who have completed the week-long training delivery qualification course or the Master Trainer qualification course. Optimal delivery includes at least one trainer with a clinical background.

R2MR has been implemented and explicitly tailored to meet the needs of other public safety professions (PSP) in Canada, including police and correctional services (Mental Health Commission of Canada, 2017). Most recently, the Department of National Defence created a customizable version of R2MR designed specifically to support PSP that is not only freely available but is also entirely accommodated to the diverse challenges persons in public safety professions face (Canadian Institute for Public Safety Research and Treatment, 2020).^1^ Nevertheless, whatever variant of R2MR is used and the evidence-based foundations steering the content and direction of the program, little research has evaluated the effectiveness of R2MR to meet its intended goals or the felt impact it leaves on PSP (Carleton et al., 2018a, 2018b).

Carleton and colleagues’ (2018a, 2018b) study of 147 Canadian police agency employees who completed a single session of R2MR training found significant reductions in stigma post-training, as many participants reported experiencing changed attitudes and improved communication about mental health. In their study of 276 Canadian Armed Forces recruits, Frank and colleagues (2021) found that participants receiving R2MR training improved their mental health self-efficacy, which refers to a person’s beliefs surrounding their competency and capacity to achieve a good mental health outcome (Bandura, 1997). In a similar study of Canadian Armed Forces military recruits, Fikretoglu and colleagues (2019) did not find significant beneficial outcomes for psychological functioning, resilience, and military performance. Still, they discovered small but positive outcomes for mental health service use attitudes, intentions, and behaviours.

To build on this limited body of literature, in the current study, we analyze 307 open-ended survey responses by 124 Canadian provincial and territorial correctional workers to four questions posed to understand worker impacts and perceptions of R2MR training. Our study will be of interest to researchers and organizations examining mental health in the workplace, particularly in correctional settings.

## Contextualizing correctional work: mental health, risk, vulnerability, stigma

Correctional workers are regularly exposed to and must navigate a wide range of potentially psychologically traumatic events (PPTEs) and diverse stressors within their occupation (Ricciardelli, 2019; Ricciardelli et al., 2018, 2020a, 2021, 2022). As shown in a recent study on PSP, 11 out of 16 types of PPTE were experienced by a PSP (Carleton et al., 2019), and these exposures could negatively affect PSP’s mental health (Dowden & Tellier, 2004). Mental health disorders prevail among PSP, including correctional workers, where, in Canada, about 44.5% of PSP screened positive for one or more mental health disorder(s) and 55% of correctional workers screened positive for one or more mental health disorder(s) (Carleton et al., 2018a, 2018b). Increased mental health risks in correctional worker populations and their workplace, in addition to the cultural stigma attached to having a mental health disorder, invigorated the importance of using workplace training programs like R2MR.

Stigma, referring to an ideology or theory, is how an attribute can leave one discredited or discreditable due to how audiences interpret the attribute (see Goffman, 1963). A discreditable stigma (e.g., that of an invisible attribute) can emerge among correctional workers who too often are already struggling with mental health disorders, which can prevent them from seeking help or disclosing their mental health status for fear of being “discreditable” (Ricciardelli et al., 2018). Losing creditability in their occupational role—due to cultural designations such as being lazy, deceitful, or simply not fit for the job—constitutes an undeniable barrier to treatment-seeking that is only compounded by the fact that recognizing one is suffering from mental health challenges is not always germane to individual experiences. Too often, individuals will not seek treatment, engage in self-care, or take time off from work when experiencing compromised well-being due to such negative associations with their health status, particularly if they admit to experiencing compromised mental health (Karaffa & Koch, 2015; Lyons et al., 2017).

Recognizing that the stigma of mental health impacts care seeking (Clement et al., 2015; Corrigan et al., 2014; Karaffa & Koch, 2015) and increases the risk of being viewed negatively (e.g., perceived as dangerousness or unreliable; Crisp et al., 2000; Hinshaw & Stier, 2008; Lyons et al., 2017; Penn & Martin, 1998), R2MR was developed to draw attention to and “normalize” mental health. In the current study, we seek to unpack the effectiveness of R2MR in reducing stigma, in motivating help seeking, and, quite simply, to understand how persons conceptualize exposure to R2MR training. We recognize that as a program, R2MR can be tied to structural stigma, which is often inherent to institutional or system-level processes, and may encourage the identification and recognition of persons who are then stigmatized (Lyons et al., 2017; Ricciardelli & Moir, 2013). In referring to structural stigma, Ricciardelli and colleagues (2018) write that “engaging in programming designed to reduce symptoms of a mental disorder systematically indicates to any observers that participants have mental disorders”. Thus, we also question how R2MR and structural stigma interrelate. Overall, evidence is mounting that suggests PSP are challenged by mental health disorders (Carleton et al., 2017; Ricciardelli et al., 2018); as such, consequences from associated stigma can be detrimental, causing personal, community, and economic hardship. Given the movement and momentum to introduce mental health awareness and training across correctional services, our study is internationally relevant and applicable, expanding discourses engaging with consequences of PPTE exposure that are, beyond universally experienced, inherent to correctional work.

## Methods

### Materials

Our data is drawn from the Correctional Worker Mental Health and Well-being Study, which was an anonymous, confidential, and voluntary survey administered to correctional workers in the Canadian provinces of Manitoba (MB), Saskatchewan (SK), Nova Scotia (NS), New Brunswick (NB), Newfoundland and Labrador (NL), and the Yukon Territory (YT), between 2018–2020, prior to the onset of the COVID-19 pandemic. In the current study, we analyze survey responses to four open-ended items eliciting feedback on the participants’ experience, recollection, learned skills, and perspectives on R2MR training: (1) “What did you find most helpful about R2MR training?; (2) Can you provide examples about what you remember about R2MR?; (3) What skills will be the most challenging to implement and why?; and (4) What skills will be the easiest to implement and why?

### Participants

A total of 1,999 correctional workers participated in the broader survey, including correctional and probation officers, program officers, nurses, rehabilitative staff, other healthcare staff, administrative staff, managers, teachers, and other employees within the community, administrative, and institutional correctional services. Of the 1,999 correctional workers who participated in the survey, 98, 60, 51, and 98 responses were recorded for questions one, two, three and four respectively, equaling 307 total responses to the questions under study. Of the 307 total responses, 45 originated from Manitoba, 13 from Saskatchewan, 205 from Nova Scotia, 9 from New Brunswick, 30 from Newfoundland and Labrador, and 5 from the Yukon Territory. Overall, these survey responses were generated by 124 unique participants. Sample size discrepancies are partly due to the differences in the size of each jurisdiction. We have chosen not to share any further demographics about participants due to anonymity concerns since some of the jurisdiction sample sizes are quite small.

### Procedure

Research ethics boards at the University of Regina and Memorial University of Newfoundland approved the study. Respondents were recruited with the aid of a membership listserv and representatives from the Canadian Ministry of the Solicitor General or Department of Justice/Public Safety and/or the local union. If desired, respondents could complete the survey during paid work time or forward the email containing a link to the survey to their personal email and complete the survey outside of work hours in a setting of their choosing. A randomly generated access code was provided to each participant, enabling respondents to log in and out of the survey and complete the survey in multiple sittings, if needed. The average length of time to complete the survey was around 40 min. Responses varied in length, ranging from a few words to lengthy, illustrative examples and commentary.

### Analysis

Our analytic process employed a constructed semi-grounded emergent theme approach (Charmaz, 2014; Glaser & Strauss, 1967; Hesse-Biber & Leavy, 2003). We analyzed the data by coding each participant’s words first into ‘parent’ (e.g., primary themes) and then ‘child’ (e.g., secondary themes) and ‘grandchild’ (e.g., tertiary themes) nodes based on emergent themes in QSR NVivo. As the analytical process was inductive, we did not know what themes would emerge from the data. As such, we framed the study based on what the data revealed theoretically but did not create any theory; hence, we call our analytical approach semi-grounded (see Ricciardelli et al., 2010) since many ‘fuller’ approaches to grounded theory methodology aim for theory construction.

Though perhaps difficult to distinguish between semi-grounded and thematic approaches to data analysis, our approach differs from inductive thematic analysis because the coding and analytical processes for grounded theory are on-going throughout data collection, whereas thematic approaches to data analysis – though ‘flexible’, adaptive, and overlapping with a variety of techniques and paradigms – tend to follow a more linear process that waits until all data are collected before generating, developing, reviewing, refining, and defining themes (Braun & Clarke, 2021). We approached data analysis as a team and analyzed data during many phases of data collection, writing, and analysis. For example, the first author reviewed the preliminary and on-going coding completed by the research team to fully immerse in all of the data, and to allow more nuances to emerge from the data. The data were then combined from the items under analysis into one file that distinguished responses across all provinces and the territory for ease of analysis and we found no discernible differences between participating jurisdictions. Working with the collective data set allowed us to gather a sense of the whole data and the key themes across responses (Corbin & Strauss, 2015), as well as help us make decisions regarding how to best represent the key themes.

We selected quotes in the presentation of results that emphasize respondent voices but also ensures we only use quotes that do not identify the respondent or impinge on their privacy. We completed minor edits of the data quoted to assist with readability, flow, and correct grammatical and spelling errors, making sure to never compromise interpretations, meaning, or tone. Although we recognize the complexity and tension in trying to quantify themes in qualitative research, we have provided percentages of our respondents contributing to identified themes since recording these allowed us to better ascertain thematic data saturation and provide readers with a glimpse of the most predominant themes found in our data (Guest et al., 2020).

## Results

We asked participants four open-ended questions regarding their perspectives and experiences with R2MR training. The results section is thus divided into four parts and presents the most predominant themes and other nuances found in response to each question. First, we discuss what participants found most helpful about R2MR training. Second, we show what participants recollected from their training. Third, we identify the learned skills respondents believe will be the most challenging to implement in practice. Lastly, we discuss the learned skills respondents believe will be the easiest to implement.

### What did you find most helpful about R2MR training?

In total, 12 of 98 respondents (12%) discussed explicitly how the objective of the R2MR program of de-bunking and reducing mental health stigma in public safety professions was the most helpful aspect of their training. Respondents wrote, for example, “reduce stigma” (MB48); “handling stigma and debunking myths” (SK483); “overcoming the stigma of mental health to ask for help” (NS22); and “reducing the stigma surrounding mental health” (NS73). These participants all acknowledge that mental health stigma in Canadian correctional work remains predominant (Johnston et al., 2021, 2022; Ricciardelli, 2020b) and often serves as a “huge barrier to why people do not reach out” (NS171). However, participants believe R2MR training works to reduce stigma by facilitating “open discussions” (YT18) and “open communication” (NS33) about mental health and by providing staff with resources and strategies to “handle” or “de-bunk” (SK483) stigma.

NS112 further extrapolates the positive implications of an increasingly open dialogue about mental health in the workplace through a training initiative in writing, “I learned that I was not alone. Made me feel better about myself. Learned different ways to deal with mental illness”. NS112’s words reveal how bringing together colleagues to discuss and learn better strategies for dealing with mental health concerns, stigma, and barriers formed a community – a space where they could understand that other workers around them may be dealing with similar challenges, and thus could work together to help one another cope or “feel better.” In some cases, R2MR and the newly formed community helped correctional workers understand more about their mental health struggles. For example, NS36 realized how the training “helped [them] recognize [they] had situational anxiety.” NL69 exclaimed that “it [R2MR] saved my life”, which emphasizes how R2MR training, by virtue of fostering open dialogue about mental health, can encourage correctional workers receiving the training to speak out about their concerns, who, prior to completing it, may have been struggling alone.

Another 13 of 98 respondents (13%) explained the most helpful aspect of R2MR training, beyond better recognition of their own mental health status or “need for self-care” (NS12), was the specific resources and techniques R2MR provided to help identify problems emerging in their colleagues, incarcerated people, or others around them more generally. For instance, respondents wrote of how R2MR helped them to develop “understanding, recognizing when someone may need support” (MB63) or “symptoms of mental illness; ways to approach a patient of mental illness and possible helpers to reach-out to” (MB244). Further, respondents felt able to “better recognize mental health issues in co-workers and deploy better strategies to discuss these issues” (NS44) and had “training in recognizing mental health disorders in offenders” (NS130); “I found it helpful when dealing with offenders and with co-workers” (NB19). The training helped with the processes of “recognizing signs and acceptance of those whom are in distress” (YT31). Consistent across these excerpts is how adequate mental health extends beyond simple recognition of a problem; as such, R2MR training provides proactive strategies to help correctional workers respond to emerging issues in others and helps workers better “understand” and “accept” people experiencing mental health problems.

Five of 98 (5%) respondents specified in detail the skills, resources, or networks they found most helpful from their R2MR training. These include techniques of de-escalation (e.g., “learning how with the power of words to make a bad situation calm” (NS18)) and resources for support: “list of contacts to offer as direction to individuals in crisis” (NS58); “I received a list of places I could get help if need be” (NL65)). Here, NS18 references how sharing a few encouraging and impactful words with someone experiencing a “bad situation” in relation to their mental health can make great strides toward de-escalating the situation. NL65 then cites that receiving a list of resources they can access in the event of experiencing distress, a mental health need or vulnerability was also helpful. Additional skills valued were “learning to talk with people who have mental health issues” (NS62) and “talking about support networks and how many people suffer” (NS71). NS62 expresses how communication skills were learned through R2MR, while, perhaps more implicitly, NS71 iterates that learning how to build support networks not only can lessen the impact of adverse mental health, but may also help workers realize just how often correctional workers suffer as a consequence of their occupational roles, responsibilities, or other life stressors.

### Can you provide examples about what you remember about R2MR?

In R2MR training, instructors teach participants how to identify their mental health status on a continuum model comprised of “healthy, reacting, injured, or ill” and “The Big 4” actions to take at each phase of the continuum (“goal setting,” “positive self-talk,” “visualization,” and “tactical breathing”; Mental Health Commission of Canada, 2017). In total, 15 of 60 respondents (25%) identified recalling the mental health continuum and how best to navigate the continuum. To exemplify the former, respondents recalled the “Mental Health Continuum” (MB48) and “The Chart mostly of what to look for in people around you and where they fall into the different stages (NS74)”. Regarding the latter, respondents wrote “goal setting, positive self-talk, focusing drills” (SK295), “healthy coping strategies, Big 4” (NB19), and “tactical breathing, visualization” (NL13). Though 75% of respondents (45) did not indicate recalling the chart, it is important to clarify that this finding does not mean respondents did not necessarily derive meaning from the mental health continuum chart but may have used the space in the survey question to simply elaborate on other items of recall. Thus, we still argue these survey responses demonstrate the effectiveness tied to the presentation of mental health identification in a simple and easy-to-visualize chart that articulates clearly the ways people can move around a complex continuum aimed at identifying their mental health status.

The value and necessity of peer support was another recalled theme 6 of 60 respondents (10%) identified. For example, a selection of respondents wrote “we are in it together, we all share experiences to help one another” (M442); “Importance of peer support” (NS29); and “there are peers at work that you can contact for support, many suffer from mental illness” (NS71). These excerpts demonstrate how staff can complete R2MR training feeling like a team, often because of the power of sharing experiences and acknowledging the prevalence of mental health problems among correctional workers. Moreover, when looking at the situation positively, many correctional workers can offer peer assistance, help, resources, and above all else – reciprocity, empathy, and mutual understanding.

Alongside developing camaraderie and promoting peer assistance, 16 of 60 respondents (17%) recalled the skills they developed tied to how best to respond to someone experiencing mental distress or crisis. Participant MB489 wrote: “how to respond to someone who is suffering and how to recognize someone who is struggling,” while NS51 described learning “how to identify triggers and solutions for potentially provoking crisis situations” (NS51), while others, like YT18, noted learning “crisis intervention.” Given the inevitability that front-line correctional workers will encounter colleagues, prisoners, or visitors in distress, these respondents learned techniques to first recognize and then improve intervention strategies. Some respondents identified precisely different ways to approach someone in distress, like NS90, who wrote learning to identify “signs of those in distress and how to use a calm voice to talk to them, do not try to touch someone in distress in most cases.” NS692 described learning how to “show empathy towards the person and let them talk when they need to and not to judge them.” Such techniques demonstrate the value of employing a caring orientation when interpreting and approaching those in mental distress. In addition, skills taught counter mental health stigma by treating those in crisis as people in need who correctional workers should listen to and not judge, given their state.

An additional 14 of 60 respondents (13%) discussed how exactly R2MR training worked to combat mental health stigma, such as NS64, who wrote, “leader described being stabbed on the job. Got over his own stigma”. In this case, listening to a trainer and colleague discuss their own personal experience with violence, mental health injury, and subsequent battle with stigma provided the correctional worker with a real-world example of how one can reach a positive end in their mental health journey, and then use their experiences to help others. NS91 also wrote, “we learned how to realize we have mental health problems and what resources are out there for us to access. And we learned that there should not be a stigma about mental health”. Here, NS91 came to the realization that correctional workers are disproportionately (and often inevitably) struck by mental health suffering and injury, but there are treatment and other resources available to them that, when accessed, should never produce feelings of shame, guilt, embarrassment, or stigma.

In summary, these excerpts show the lasting impact R2MR training can have on correctional workers who, by and large, were able to recall many skills learned and how peers can all work to improve workplace mental health.

### What skills will be the most challenging to implement and why?

In total, 14 of 51 respondents (27%) discussed the specific skills learned during their R2MR training that would be the most difficult to implement. Muscle relaxation, for instance, was described as hard to engage in at work, as MB48 stated, “progressive muscle relaxation—hard to do effectively at work. Better for helping me sleep”, and MB57 wrote, “progressive muscle relaxation because I lose my focus and feel uncomfortable with it.” Engaging a relaxation or tactical breathing technique requires concentration for effective employment, and many correctional workers may face challenges doing so when confronted with a highly dangerous or volatile situation at work, such as prisoner violence or self-injury. NL82 elaborated on the trickiness of using de-escalation skills during scenarios that require quick-thinking and action: “Deescalating volatile situations,” and NS9 supported this position in stating that “deescalating someone as I would be afraid of saying the wrong thing.” Though these learned skills are effective in theory, NL74 claimed that “skills have to be practiced” to be understood and implemented well, and that their organization does “not giv[e] enough refresher training,” which may make it difficult for some correctional workers to remember and sustain these skills.

In recognition that mental health stigma still permeates many public safety professions, 8 of 51 respondents (16%) expressed, for a number of reasons, that reducing and eliminating stigma in the workplace would be the most challenging task. Here participants wrote that “recognizing and responding as there is a stigma to most of this” (MB231); “breaking down the stigma” (MB435); “getting help, stigma still exists and I don’t trust people to tell them my issues” (NS71); “society to overcome stigma” (NL16). MB231 and MB435 both recognize the work required to break down stigma in their profession, while NS71 and NL16 point out that the problem is entrenched at both the individual and societal levels. More specifically, NS71 identifies how stigma prevents the formation of trust toward colleagues and thus reaching out to peers, perhaps because respondents fear that opening up will back-fire and trigger negative consequences, even though, as discussed earlier, their colleagues may know, understand, and appreciate what they are managing more than anyone else. As NL16 emphasizes implicitly, stigma at the institutional level is influenced by broader societal stigma, and thus the problem is not localized and operates in conjunction with social life and mental health discourses functioning and spreading outside of prison settings.

Another 14 of 51 respondents (27%) described emergent difficulties in trying to reach out to colleagues who may be presenting a mental health problem, or experiencing the challenges associated with identifying and responding to problems emerging in themselves. For instance, MB489 wrote “Having success as all parties have to be onboard and follow through. Trying to have someone talk is difficult if they’re not willing.” NS21 describes “the buy in of staff” as a challenge, while NS22 put forth that “seeking help, not everyone is ready to accept help.” Others, like NS42, described how “getting help or talking to someone” was a barrier or how “it is challenging to recognize when I’m getting stressed to the point of needing to seek mental health support” (NB19). Across excerpts, evidenced is the realization that unless staff become more willing to put into practice R2MR’s efforts to create open, non-judgmental dialogue around mental health and foster an environment that encourages struggling staff to seek help when signs of decline or distress appear, then little change can occur. NS59 further identifies that viewing mental health as a “right” rather than a privilege or an individual problem is a necessary shift in correctional services that will produce better mental health outcomes, as NS171 elaborates, “helping ourselves is our last option most times, we don’t see it as viable or worthy and perhaps that is entrenched in the stigma of mental health.”

Collectively, responses reveal the strong capacity of respondents to identify practical challenges associated with implementing skills learned under R2MR’s training regimen, but alongside the assertion that they are all valuable skills nonetheless that are realized best in caring, non-stigmatized workplace cultures and society.

### What skills will be the easiest to implement and why?

Here, 35 of 98 respondents (36%) indicated that changing workplace culture by exercising compassionate, empathetic, and open communication, listening, and social skills would be the easiest change to implement from their R2MR training. NS13 explains “just relating to people” as easy, while “sharing my experience with mental disease to help others” (NS22) and to “try to have more understanding and empathy” (NS57) were also noted, suggesting supportive communication was possible among correctional workers. This was further reinforced by NB19, who felt, “It will be easiest to implement being a part of a supportive environment and promoting positive mental health.” The key to such an environment is “listening” (Y18), as NL84 explains that “listening, cause its most important.” Overall, underpinning these responses is the willingness of correctional workers to be the change they want in their workplace, which they can demonstrate simply by “car[ing] about [their] co-workers” (NS68) and taking the time to make their workplace as supportive of an environment as possible, that includes hearing their peers. NS71 believed they can easily “talk about situations with coworkers because they understand what we go through,” which again, emphasizes the positive relationality and relatedness among staff that R2MR seeks to mobilize in public safety workforces.

Easy-to-implement techniques learned from training were also identified by 32 of 98 respondents (33%). These techniques include: “thought stopping because I now use it many times a day to control racing thoughts and negative self-talk” (MB57); “tactical breathing and self-talk. Can do these anywhere” (MB48); “breathing is easy to use for beginners” (NL11); “active listening, using a calming tone” (NL82); “recognizing persons in distress. Assessment skills are improving” (YT13). These excerpts reveal how, with education and practice, R2MR can provide correctional workers with techniques to help reduce their adverse mental health challenges and improve their awareness, which can be used practically anywhere and across varying contexts. This is important, especially for newer employees who may require easier skills to engage until they gain more experience and practice using the more difficult skills and assessment techniques taught.

SK295 also emphasized the importance of being realistic about one’s expectations of the R2MR training: “goals, breaking them down into realistic goals with checks and balances to not disappoint oneself,” which shows the reflexivity and self-assessment needed throughout the process to determine what skills are working and what are not. NS29 recognized that “self-care” is the easiest skill to implement because it does not “require anybody else’s help.” These words re-emphasize how mental health is a complex, team-effort, and that sometimes individual efforts to reduce adverse mental health outcomes can be easier to engage when one is unsure how to relate to their colleagues and social environment. This resonated particularly in NS50’s words: “believing people when they disclose information,” which again troubles discourses of suspicion in relation to staff bringing forward their mental health struggles, conditions, and illnesses. That said, NS73 recognized that change is happening, albeit gradually in some environments, because “we are all learning more about mental health growing up,” and thus, with more education, resources, and effort, corrections work in Canada will better facilitate good mental health and wellness.

## Discussion

R2MR is intended to increase mental health awareness and, thus, improve the mental health-related behaviours of trained PSP. As such, our data elucidate how R2MR is interpreted, and what R2MR’s role could be in increasing mental health awareness. Participants explained that R2MR reduced stigma and debunked myths about mental health, thus increasing awareness. Nevertheless, stigma in correctional work remains a barrier to treatment-seeking and mental health awareness (Ricciardelli et al., 2018, 2020b). R2MR, however, has a role in increasing discussion about mental health in correctional services, creating a space for dialogue, which is likely the first step toward increased recognition of mental health needs and, thus, treatment-seeking. Some respondents felt that R2MR training was a form of personal release – a starting point to acknowledge that something was not quite the norm in terms of mental health and help was required. Moreover, R2MR was considered a knowledge channel that provided a space to recognize if one personally, a colleague, or even a criminalized person required mental health support—said differently, R2MR helped participants understand and accept how people experience their mental health.

Respondents described learning skills in R2MR training, including how to relate to people and provide a listening ear during times of crisis. They recalled the mental health continuum and elements of the program – from breathing to visualization to self-talk. Overall, these respondents demonstrated how many R2MR participants were able to identify positive and helpful components of the training regimen, which they could apply to their work and personal lives—always with the aim of improving their workplace mental health framework and practices, as well as normalizing conversations around mental health. Reports from the Canadian Armed Forces indicate that the R2MR program does reduce stigma and improve treatment-seeking practices and self-care (Blacker et al., 2018). Other results of multilevel analyses showed that R2MR training might lead to at least a slight reduction of mental health stigma, but for further and sustained reductions, refresher trainings may be needed (Carleton et al., 2018a, 2048b, 2020). This evidence shows an association between some training programs like R2MR and lower possibilities of mental health disorders. However, inconsistency exists (Fikretoglu et al., 2019); R2MR seems to reduce mental health stigma but does not necessarily lead to changes in mental health symptoms, so more research on this matter is warranted.

Respondents felt that certain learned skills and elements of peer support were difficult to implement, for instance, muscle relaxation or tactical breathing while on shift, some forms of de-escalation, and the ability to reach out to colleagues appearing to be in mental distress. What is clear here is that the stigma of mental health prevails such that persons are unable to comfortably, and with confidence, approach colleagues who they feel may have compromised mental health (Corrigan & Rüsch, 2002; Corrigan & Wassel, 2008; Mak et al., 2007). Thus, despite the efforts and training, there appears to be a disconnect between select skills taught and what is able to be applied in the context of correctional work. Alternatively, there are some skills described as easier, even easy, to implement. These include empathy, compassion, more open communication, and simply being able to relate to others. Although appearing to be simple skills, such implementations work to change the workplace culture, which is an important factor impacting mental health and thus collective well-being.

R2MR also seemed to create a space in which respondents became more open to peer support, even finding peer support of great importance. With this finding in mind, we suggest further research into peer support, specifically what peer support should look like if peer support is to be employed within correctional services and the effectiveness of any available peer support programs available or implemented. Given the differences between R2MR and The Working Minds, we recommend future evaluations differentiate between the diverse programs and to determine the effectiveness of the specific programs. Additionally, researchers may wish to see if there are any discernible outcome differences in people trained in R2MR, The Working Minds, and the online variant of R2MR. Particularly given the impacts of COVID-19 on communication styles and the ability to gather in person, such studies are necessary to determine the impact and effect of programs to see if the online program is a possible way forward toward supporting mental health among the PSP and increasing mental health awareness.

### Strengths and limitations

In the current study, we build on emerging literature pertaining to R2MR and the greater context of mental health training programs in Canada by adding the perspectives of correctional workers who experienced the R2MR program to the dialogue. Still, our work is not without limitations. Our data are comprised of survey responses to open-ended questions presented to us anonymously by participants. As such, we could not probe respondents for any additional information or clarity about their perceptions of and experiences with the R2MR program. Future qualitative research that engages interview or focus group methods would provide additional insights, particularly when presented with opportunities to probe for clarity or more information. Due to overlap between institutional and union listservs, the precise response rate cannot be determined for this study. Given this limitation and the smaller sample sizes in some provinces and territories, as well as the fact this is a qualitative study, we do not infer generalizability, as an accurate response rate would generate further insight into the extent to which correctional workers are comfortable sharing information related to mental health training and readiness.

## Conclusion

The current study shows that although mental health stigma in the Canadian correctional workplaces remains a barrier, the R2MR program has played an important role in increasing awareness of mental health issues and teaching skills that can be used at work or other spaces to reduce the impacts of adverse mental health and improve treatment-seeking. Our results also highlight the importance of peer support and facilitating a safe communication space in public safety workforces. This safe space allows colleagues to discuss their mental health concerns regardless of cultural barriers and work together to cope with and respond to their mental health challenges.

R2MR seemed to be a helpful resource for identifying, on a continuum, the signs of those who are in distress and whose mental health is impacted. Besides that, participants felt this program educated them on developing skills to help deal with a person in crisis, whether that be a colleague, incarcerated person, or themselves. Finally, as was highlighted in our findings, the learned skills from R2MR should be practiced enough by participants to become memorable and sustained, which is best realized when organizations continue to provide adequate support and refresher training.

## Data Availability

The datasets generated and/or analyzed during the current study are not publicly available due to reasons surrounding confidentiality and anonymity.

## Footnotes

1 The National Department of Defence offers R2MR for PSP and for armed forces personnel. The Mental Health Commission of Canada offers a variant of R2MR for a fee, called The Working Minds, which was adapted from R2MR. The Canadian Institute of Public Safety Research and Treatment also offers an online version of R2MR. In this paper, given The Working Minds was prior called R2MR, we do not differentiate between the version of R2MR in which one was trained.
